# The identification of novel regions for reproduction trait in Landrace and Large White pigs using a single step genome-wide association study

**DOI:** 10.5713/ajas.18.0072

**Published:** 2018-05-31

**Authors:** Rattikan Suwannasing, Monchai Duangjinda, Wuttigrai Boonkum, Rutjawate Taharnklaew, Komson Tuangsithtanon

**Affiliations:** 1 Department of Animal Science, Faculty of Agriculture, Khon Kaen University, Khon Kaen 40002, Thailand; 2 Research and Development Center Betagro Group, Pathumthani 12120, Thailand; 3 Betagro Hybrid International Company Limited, Bangkok 10210, Thailand

**Keywords:** Genomics, Single Step Genome-wide Association Study (ssGWAS), Candidate Genes, Single Nucleotide Polymorphisms (SNPs), Swine

## Abstract

**Objective:**

The purpose of this study was to investigate a single step genome-wide association study (ssGWAS) for identifying genomic regions affecting reproductive traits in Landrace and Large White pigs.

**Methods:**

The traits included the number of pigs weaned per sow per year (PWSY), the number of litters per sow per year (LSY), pigs weaned per litters (PWL), born alive per litters (BAL), non-productive day (NPD) and wean to conception interval per litters (W2CL). A total of 321 animals (140 Landrace and 181 Large White pigs) were genotyped with the Illumina Porcine SNP 60k BeadChip, containing 61,177 single nucleotide polymorphisms (SNPs), while multiple traits single-step genomic BLUP method was used to calculate variances of 5 SNP windows for 11,048 Landrace and 13,985 Large White data records.

**Results:**

The outcome of ssGWAS on the reproductive traits identified twenty-five and twenty-two SNPs associated with reproductive traits in Landrace and Large White, respectively. Three known genes were identified to be candidate genes in Landrace pigs including retinol binding protein 7, and ubiquitination factor E4B genes for PWL, BAL, W2CL, and PWSY and one gene, solute carrier organic anion transporter family member 6A1, for LSY and NPD. Meanwhile, five genes were identified to be candidate genes in Large White, two of which, aldehyde dehydrogenase 1 family member A3 and leucine rich repeat kinase 1, associated with all of six reproduction traits and three genes; retrotransposon Gag like 4, transient receptor potential cation channel subfamily C member 5, and LHFPL tetraspan subfamily member 1 for five traits except W2CL.

**Conclusion:**

The genomic regions identified in this study provided a start-up point for marker assisted selection and estimating genomic breeding values for improving reproductive traits in commercial pig populations.

## INTRODUCTION

The genetic improvement is one approach to improve reproductive performance. However, the reproduction traits are characteristic of low heritability and difficult using conventional selection method to improve. The conventional selection method may provide a lower accuracy, in comparison to the whole genome selection [1]. In the current development of molecular technique, such as single nucleotide polymorphism (SNP) chip has been widely used in for genome-wide association study (GWAS) to be a powerful tool in the identification of genomic regions or quantitative trait loci (QTL) related to an economically important trait. Single-step GWAS (ssGWAS) is the new GWAS approach which utilized all data (genotypes, phenotypes, and pedigree information) jointly in one step, proposed by Wang et al [2]. This approach can use for many models and computing is fast and simplicity [2]. In pigs have been GWAS study using SNP chip in reproduction traits especially litter trait such as the number of born alive (NBA), total number born, mummy (MUM), stillborn (SB) and total litter birth weight [3,4]. While, no previous literature has studied the GWAS of pig weaned per sow per year (PWSY), litter per sow per year (LSY), pigs weaned per litters (PWL), born alive per litters (BAL), non-productive day (NPD), and wean to conception interval per litters (W2CL). Therefore, finding the genomic regions and candidate genes in the regions of significant SNPs from the whole genome that related to the reproduction trait can be used as a powerful tool for selection to obtain the high reproductive performance. The objective of this study was to investigate a GWAS for identifying novel regions affecting on the reproduction trait in Landrace and Large White pigs.

## MATERIALS AND METHODS

### Animal care

Institutional Animal Care and Use Committee (IACUC) approval was not obtained for this study because the data were obtained from an existing database on pig breeding.

### Animals and data

The data used for this study collected from Thailand commercial herds; extracted from the SowTracker (Version 3.4.7) reproductive data management software. The number of animals considered in the analyses was different for each trait because of the lack of data for some animals. A total of 11,048 Landrace and 13,985 Large White pigs were collected data records with 13,351 and 16,731 pedigree records respectively. These sows were raised in six farms. The reproduction traits included PWSY, LSY, NPD, W2CL, BAL, and PWL were recorded to a maximum of 10 parities between 2006 through 2015. PWSY was calculated as litter per sow per year multiplied by pigs weaned per litter; LSY was calculated as (the number days of gestating divided by 115 days) divided by (the number of days in the breeding herd divided by 365 days); W2CL, BAL, and PWL were collected on every litter and divided by total litters; the NPD was calculated as 365 minuses productive days multiplied by LSY, productive days was the total number of days that all gilts and sows were either gestating or lactating. Then checks the normal distribution before basic statistical analyses such as mean, standard deviation and coefficient of variation.

### Genotype data and quality control

Used 321 animals; 140 Landrace and 181 Large White pigs were genotyped with the Illumina Porcine SNP 60k BeadChip, contained 61,177 SNPs. The quality control for genotypes SNPs of each breed following criteria was: SNP call rates <0.90, genotype call rates <0.90, minor allele frequencies <0.05, Monomorphic and checks parent-progeny Mendelian conflicts were selected for further analysis. A total of 129 Landrace and 175 Large White pigs with a total of 47,590 and 47,865 SNPs respectively were available for the genome-wide association analyses in this study.

### Genome-wide association analysis

#### Single-step genome-wide association study

The genome-wide association analysis was estimated by using single-step genomic BLUP (ssGBLUP) [2]. GWAS by ssGBLUP can be called ssGWAS. In this methodology, multivariate and separate breed analyzed the data. The statistical model was used:

y=Xβ+Zu+e

Where y represented a vector of observations (PWL, BAL, W2CL, LSY, PWSY, and NPD), *β* is a vector of fixed effects. The fixed factors used in this study were last farrow-month, last farrow-year, last parity and farm, *u* is a vector of additive genetic effects, which was assumed to be distributed $\text{N}(0,\;\sigma_{u}^{2})$, e is a vector of residual effects, which was assumed to be distributed as $\text{N}(0,\;\text{I}\sigma_{\text{e}}^{2})$, X is the incidence matrix related records to fixed effects; and Z is the incidence matrix related records to additive genetic effects.

The genetic variance component was obtained by using the restricted maximum likelihood (REML) and all analyses for REML, BLUP, and ssGWAS were run by using the BLUPF90 software [5]. In the animal model, the inverse of the numerator relationship matrix (A^−1^) was replaced by H^−1^ that combines the pedigree and genomic information [6].

$$\text{Where},\;{\text{H}}^{-1}=A^{-1}+\left[\begin{array}{cc} 0 & 0\\ 0 & {\text{G}}^{-1}-A_{22}^{-1} \end{array}\right]$$

Where G^−1^ is the inverse of the genomic relationship matrix and $A_{22}^{-1}$ is the inverse of the pedigree-based relationship matrix for genotyped animals. The G matrix is a genomic relationship that constructed weighting each SNP effect by its expected variance in an iterative procedure can be created by following [7] as:

$$G=ZDZ^{\prime }q$$

Where *Z* is a matrix relating genotypes of each locus (0, 1, or 2) adjusted for allele frequencies, *D* is a diagonal matrix of weights for variances of SNP effects (initially *D* = *I*), and *q* is a weighting factor. The weighting factor ensuring the average diagonal in *G* which is close to that of *A_22_* [8]. The SNP effects and weight for ssGWAS can be derived as follows [2]:

- Let *D* = *I* in the first step and calculate *G* matrix; *G* = *ZDZ’q*
- Calculate GEBVs for the entire animal in the data set using ssGBLUP.
- Convert GEBVs to SNP effects (*û*): *û* = *qDZ’*[*ZDZ’q*]^−1^*â_g_,* where *û* is a vector of SNP marker effects, and *â_g_* is the animal effects of genotyped animals.
- Calculate weights for variances of SNP effects: $d_{i}={\hat{u}}_{i}^{2}2P_{i}(1-P_{i})$, where *d_i_* is the genetic additive variance by each SNP marker, ${\hat{u}}_{i}^{2}$ is the square of the i-th SNP marker effect, *P_i_* is the allele frequency of the second allele of the ith marker in the current population [9].
- Normalized SNP weight to remain the total variance constant.
- Calculate *G* matrix; *G* = *ZDZ’q*
- Exit or Loop to step 2

The iterative process was repeated two times from step 2 to 7, and the percentage of genetic variance explained by i-th consecutive SNPs (SNP window) was calculated as described by Wang et al [2]:

$$\frac{Var\;(a_{i})}{\sigma_{a}^{2}}\times 100\%=\frac{Var(\Sigma_{j=i}^{5}Z_{j}{\hat{u}}_{j})}{\sigma_{a}^{2}}\times 100\%$$

Where, *a_i_* is the genetic value of the i-th region that consists of consecutive 5 SNPs, $\sigma_{a}^{2}$ is the total genetic variance, *Z_j_* is a vector of the gene content of the j-th SNP for all individuals, and *û_j_* is marker effect of the j-th SNP within the i-th region.

In this study used 5 SNP window because it has been reported by Beissinnger et al [10] that both sliding windows of 5 or 10 SNPs had the most favorable ratio of detection rate to false-positive rate than larger window sizes.

### Candidate gene search

The consecutive SNPs which explained 1% or more than of genetic variance were selected as SNPs that considered to associated with reproduction traits. These regions were used to determine possible putative QTL or candidate genes based on the regions those within the gene. Gene search was carried out by using the Sus scrofa Build 11.1 assembly database release on August 2017 and GeneCards (https://www.genecards.org/) for identification of biology function of the associated genes. In this study used HGNC gene symbols referenced from the HUGO Gene Nomenclature Committee. If the genes were not found in these regions, then it was considered flanking regions about 2.0 Mb upstream or downstream of QTL regions to possibly represent the locus [11]. Previously identified QTL in the pig genome was evaluated by using the PigQTLdb (http://www.animalgenome.org/cgi-bin/QTLdb/SS/index) [12].

## RESULTS AND DISCUSSION

This study is preliminary of research using genomic information to apply for field data in commercial pigs of Thailand and need to beware of using small data size. However, we use ssGWAS because this approach utilizes all available information jointly in one step and has been validated using field data which more precise estimates of variance components by including non-genotyped animals if the number of genotyped animals is limited [13].

### Genetic parameters estimation

Heritabilities calculated from the variance components are shown in Table 1. The estimated of heritabilities were low to moderate for all traits, ranging from 0.07 to 0.25. The results indicated that Landrace pigs had a heritability of six traits slightly lower than Large White pigs. The heritabilities of PWL, BAL, W2CL, PWSY, LSY, and NPD were 0.09, 0.12, 0.08, 0.17, 0.13, and 0.18, respectively in Landrace. Meanwhile, heritabilities in Large White pigs were 0.12, 0.14, 0.07, 0.25, 0.18, and 0.25, respectively. The genetic and phenotypic correlation estimates for reproduction traits are given in Table 2. The genetic correlations between NPD and the five reproduction traits in Landrace and Large White were −0.25 and −0.26 for PWL, −0.13 and −0.08 for BAL, 0.35 and 0.53 for W2CL, −0.99 and −0.99 for LSY, −0.67 and −0.65 for PWSY. The phenotypic correlation between NPD and PWL, BAL, W2CL, LSY, and PWSY were 0.06, 0.04, 0.35, −0.99, and −0.35, respectively in Landrace. For Large White the phenotypic correlation between NPD and PWL, BAL, W2CL, LSY and PWSY are 0.08, 0.05, 0.39, −0.99, and −0.31, respectively. Which, the phenotypic correlation of NPD and PWL and BAL has low magnitudes.

### Genome-wide association study

**Figure 1:**
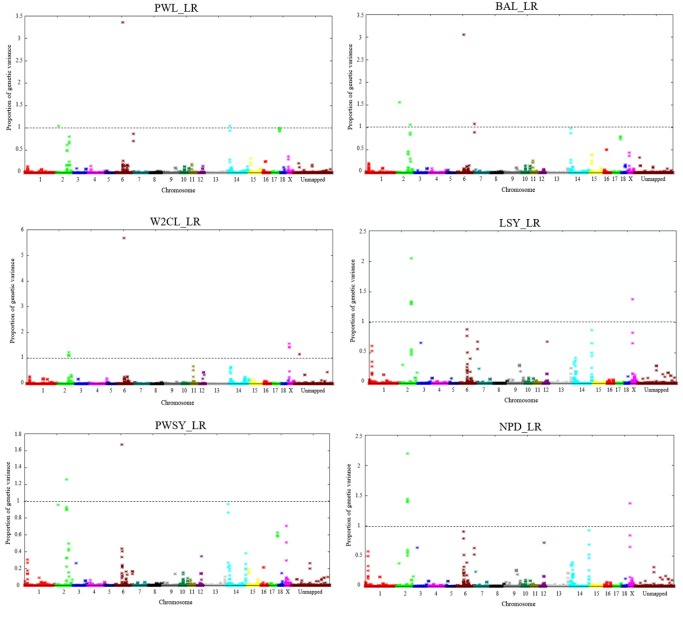
Manhattan plot of genetic variance contributed by an SNP window of 5 consecutive SNP for reproduction traits in Landrace pigs. SNP, single nucleotide polymorphism.

**Figure 2:**
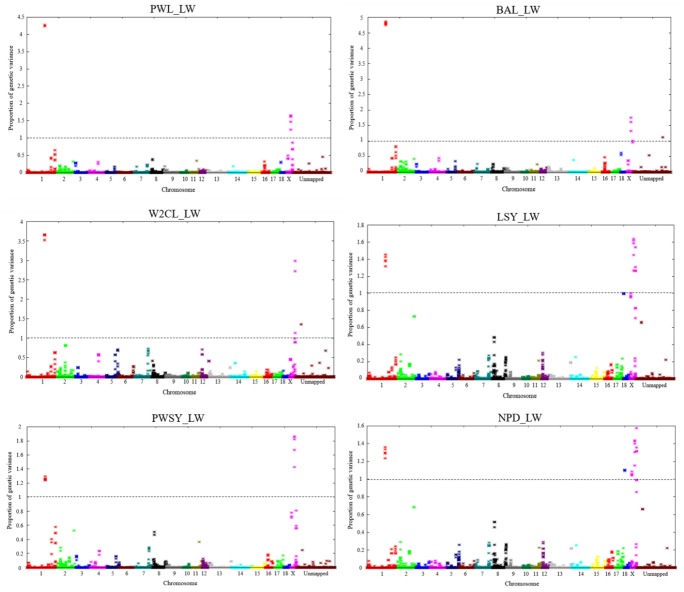
Manhattan plot of genetic variance contributed by an SNP window of 5 consecutive SNP for reproduction traits in Large White pigs. SNP, single nucleotide polymorphism.

The ssGWAS results of the 6 measured traits of Landrace and Large White were shown in Figures 1, 2, respectively. Figure 1 and 2 showed the plots of genetic variances explained by each 5-SNP sliding windows showed in a Manhattan plot. Different shades represented SNP on a different chromosome from *Sus scrofa* chromosome (SSC)1 (left) to X and unmapped (right). In total, there were 47,590 and 47,865 regions in Landrace and Large White, respectively.

Genomic regions were found to be associated with reproduction traits for Landrace and Large White pigs in Table 3 and Table 4, respectively, together with the candidate genes and associated SNPs within each region. In PigQTLdb has been reported QTL effect on reproduction traits, 144 QTL were identified for NBA and 4 QTL for the number of weaned (November 2017). Our study had detected the new genomic regions for pig reproduction traits which did not overlap with QTL intervals previously reported from PigQTLdb (http://www.animalgenome.org/cgi-bin/QTLdb/SS/index).

For Landrace pigs, the QTL regions were identified on SSC 2, 6, 14, and X. A total 60 regions were significantly associated (SNP windows that explained more than 1% of genetic variance) with reproduction traits for all six traits which were included in 11, 14, 13, 8, 6, and 8 regions for PWL, BAL, W2CL, LSY, PWSY and NPD, respectively. When the consideration of overlapped of QTL regions from all traits, it was found that a total of 25 SNPs was associated with reproduction traits. The candidate genes which had the highest of genetic variance of 5 adjacent SNPs for each trait were retinol binding protein 7 (*RBP7*) and ubiquitination factor E4B (*UBE4B*) gene located on SSC6 for PWL (3.36%), BAL (3.06%) W2CL (5.69%) and PWSY (1.67%); solute carrier organic anion transporter family member 6A1 (*SLCO6A1*) gene located on SSC2 for LSY (2.05%) and NPD (2.20%). From the results, some regions and genes showed a significant associated with more than one trait within a breed. It means that the presence of a variant may affect the multiple traits. For instance, the region that strongly associated with PWL, BAL, W2CL, and PWSY was rs81320475. The *RBP7* and *UBE4B* gene have highly associated with four traits were PWL, BAL, W2CL, and PWSY.

It was found that a total of 11 genes (located on SSC2, SSC6, SSC14, and SSCX) associated with reproduction traits in Landrace pigs (Table 5). All of them, *SLCO6A1*, *RBP7*, and *UBE4B* have the highest percentage of genetic variance and rather cover associated with all reproduction traits. The *SLCO6A1* is organic anion transporting polypeptide family, located on SSC2, which associated with W2C, LSY, NPD, and PSY. This gene strongly expressed in human testis [14,15] and has been identified as a cancer/testis antigen expressed in human lung cancer [15].

The *RBP7* is a member of the retinol binding protein family. The retinoids play roles in vision, growth, reproduction, and cellular differentiation beginning in early development [16]. This gene located on SSC6, which associated with PWL, BAL, W2CL, and PWSY. In the pig, *RBP7* gene has high expression in fat and higher expression in the endometrium on day 15 of estrous cycle compared to the pregnancy of day 15 [17]. According to Hu et al [18] reported the *RBP7* plays a role in regulating peroxisome proliferator-activated receptor gamma transcriptional activity and that adiponectin (AdipoQ) might be a potential downstream target of *RBP7* in the endothelium.

The *UBE4B* gene located on SSC6, which associated with PWL, BAL, W2CL, and PWSY. The vertebrate, *UBE4B* gene is also known as ubiquitin fusion degradation (*UFD2a*), homolog in yeast. In 2005, Kaneko-Oshikawa et al [19] showed that mice lacking *UFD2a* or deletion of ubiquitin enzymes effected on embryo-lethal and apoptosis in the heart. According to Zage et al [20] reported the *UBE4B* overexpression reduced neuroblastoma tumor cell proliferation which neuroblastoma is a type of cancer that found in an embryo or fetus in human.

For Large White pigs, a total of 69 putative QTL regions with in the significant regions (SNP windows explained more than 1% of genetic variance) were included 10, 11, 11, 11, 10, and 16 regions for PWL, BAL, W2CL, LSY, PWSY, and NPD respectively. When consideration of overlapped of QTL regions from all traits, it was found that a total of 22 SNPs was associated with reproduction traits. The candidate genes have the highest of genetic variance of 5 adjacent SNPs for each trait were aldehyde dehydrogenase 1 family member A3 (*ALDH1A3*) gene located on SSC1 for PWL (4.27%); leucine rich repeat kinase 1 (*LRRK1*) gene located on SSC1 for BAL (4.86%) and W2CL (3.66%); retrotransposon Gag like 4 (*RTL4*) located on SSCX for LSY (1.64%) and PWSY (1.86%). Finally, an uncharacterized gene (ENSSSCG00000022384) or the known gene was *RTL4* gene found on SSCX (1.57 and 1.44% respectively) was the highest percentage of genetic variance for NPD. This result indicated that some regions showed significant associated with more than one trait within a breed. It means that the presence of a variant may affect multiple traits. For examples, the region that strongly associated with PWL, BAL, W2CL and PWSY was rs80830052. The *ALDH1A3* and *LRRK1* gene have highly associated with all reproduction traits.

It was found that a total of 9 genes (located on SSC1, SSC18, and SSCX) associated with reproduction traits in Large White pigs (Table 5). There are five gene that have the highest percentage of genetic variance and rather cover associated with all reproduction traits. The *ALDH1A3* and *LRRK1* were found on every trait in Large White pigs, which located on SSC1. The aldehyde dehydrogenase (ALDH) family is the important enzyme for the aldehyde metabolism which plays an important role in embryo formation and development, cell proliferation and differentiation [21]. *ALDH1A3* is primarily responsible for oxidizing all-trans retinal to retinoic acid (RA) and active derivative of vitamin A (retinol) [21,22], which has been reported that *ALDHLA3* knockout in mouse suppresses RA synthesis, vitamin A-deficient fetuses and cause malformations restricted to ocular and nasal regions, which is responsible for respiratory distress and death at birth [23]. Moreover, *RBP7* and *ALDH1A3* may enhance the growth and proliferation of muscle cells, possibly early in embryonic development, and contribute to the phenotypic expression of high feed efficiency through stimulation of the Jnk pathway [24]. The information above indicated that these genes were important for reproduction traits and might be applicable to the screening of candidate genes related to prolificacy and implantation rate in pig production.

The *LRRK1* are large multidomain proteins containing kinase, GTPase and multiple protein-protein interaction domains which play a role in the regulation of bone mass in human mutation of *LRRK1* [25] and lead to osteosclerotic metaphysical dysplasia and causes a severe osteopetrosis.

The *RTL4* gene or mammalian retrotransposon transcripts also called Sushi-ichi-related retrotransposon homologue 11/zinc finger CCHC domain-containing 16 (*SIRH11*/*ZCCHC16*), located on SSCX. It is expressed in the brain, kidney, testis, and ovary in adult mice [26] but undetectable expressed in placental stages indicated no role during mouse placentogenesis [27]. The deletion of *SIRH11/ZCCHC16* gene leads to abnormal behaviors related to cognition which, including attention, impulsivity and working memory, possibly via the noradrenergic system [26].

Transient receptor potential (TRP) channels play fundamental roles in sensory biology. The short transient receptor potential channel 5 (*TRPC5*) gene, plays an important role in maintaining blood pressure stability [28], may provide manipulate the activity of key neurons involved in the regulation of energy balance and glucose metabolism [29]. In addition, *TRPC5* also associated with the weight of the biceps brachii muscle, which related to leg weakness in pigs [30]. The function of this gene was related to health which associated with reproduction traits by indirectly. In the current study, this gene located on SSCX and it was associated with PWL, BAL, LSY, PSY, and NPD trait in Large White pigs.

LHFPL tetraspan subfamily member 1 (*LHFPL1*) is a member of the lipoma HMGIC fusion partner (*LHFP*) gene family. It is expressed widely in all tissues, especially high in lung, thymus, skeleton muscle, colon, and ovary [31] but the function has not been determined.

Most of the QTL identified in this study are new genomic regions. Previously detected QTL for the total NBA has been reported at the SSC1, SSC2, and SSC6; for the number weaned has been reported at the SSC1 and SSC2 [12] which are same chromosome found in this study, but the QTL is in different locus or reported other reproduction traits such as SB, MUM, and gestation length. Meanwhile, other reproduction traits in this study have not been previously detected QTL from PigQTLdb.

The GWAS by using single-step identified twenty-five and twenty-two regions were associated with reproduction traits in Landrace and Large White, respectively. Among them, we focus on eight genes that associated with reproduction traits in both breeds which these regions located within the gene and had the highest of genetic variance of 5 adjacent SNPs. Three known genes were identified to be the candidate genes included two genes were *RBP7* and *UBE4B* for PWL, BAL, W2CL, and PWSY and one gene was *SLCO6A1* for LSY and NPD in Landrace pigs. Meanwhile, five genes were identified to be candidate genes in Large White, which associated with all of six reproduction traits included *ALDH1A3* and *LRRK1* and five traits except W2CL were *RTL4*, *TRPC5*, and *LHFPL1*.

The ssGWAS suitable for complex models like multiple traits and small size of genotypes animals because using all information (genotypes animals, non-genotypes animal, phenotypes, and pedigree information) to estimate genomic value for ssGWAS analysis which classical GWAS use only information from genotyped animals. However, the ssGWAS still weakness is cannot provide the p-value for each SNPs. Although the p-value can use the normalizing each SNP solution to a t-like statistical analysis; nonetheless, it difficult to apply to multiple SNPs and the future research may provide the level of significance.

## Figures and Tables

**Table 1:** Variance components and heritabilities for reproduction traits in Landrace and Large White pigs

Breeds	Traits	$\sigma_{a}^{2}$	$\sigma_{e}^{2}$	$\sigma_{p}^{2}$	*h*^2^
Landrace	PWL	0.18	1.78	1.96	0.09
	BAL	0.28	2.04	2.32	0.12
	W2CL	1.45	16.20	17.65	0.08
	LSY	0.005	0.022	0.026	0.17
	PWSY	1.57	10.38	11.95	0.13
	NPD	84.05	385.70	469.75	0.18
Large White	PWL	0.30	2.15	2.45	0.12
	BAL	0.44	2.55	2.98	0.15
	W2CL	1.31	15.46	16.77	0.08
	LSY	0.01	0.02	0.03	0.25
	PWSY	2.78	12.09	14.87	0.19
	NPD	115.60	343.50	459.10	0.25

PWL, pigs weaned per litters; BAL, born alive per litters; W2CL, wean to conception interval per litters; LSY, litter per sow per year; PWSY, pig weaned per sow per year; NPD, non-productive day.

**Table 2:** Genetic correlations (above the diagonal) and phenotypic correlations (below the diagonal) for reproduction traits in Landrace and Large White pigs

Breeds	Traits	PWL	BAL	W2CL	LSY	PWSY	NPD
Landrace	PWL	-	0.94	−0.38	0.23	0.89	−0.25
	BAL	0.84	-	−0.28	0.12	0.78	−0.13
	W2CL	−0.01	0.03	-	−0.34	−0.46	0.35
	LSY	−0.09	−0.05	−0.34	-	0.66	−0.99
	PWSY	0.91	0.77	−0.15	0.33	-	−0.67
	NPD	0.06	0.04	0.35	−0.99	−0.35	-
Large White	PWL	-	0.87	−0.21	0.25	0.90	−0.26
	BAL	0.83	-	−0.15	0.08	0.73	−0.08
	W2CL	0.02	0.05	-	−0.53	−0.41	0.53
	LSY	−0.10	−0.06	−0.38	-	0.65	−0.99
	PWSY	0.92	0.77	−0.14	0.29	-	−0.65
	NPD	0.08	0.05	0.39	−0.99	−0.31	-

PWL, pigs weaned per litters; BAL, born alive per litters; W2CL, wean to conception interval per litters; LSY, litter per sow per year; PWSY, pig weaned per sow per year; NPD, non-productive day.

**Table 3:** The regions of 5 SNP windows which explained >1% of genetic variance for six reproduction traits in Landrace, with a list of annotated genes

Traits	SSC	SNP position	Reference SNP ID number	Gene location1) (bp)	Candidate gene1)	% (var)2)
PWL	2	20563683	rs81291755	20,417,294 – 21,634,205	*LRRC4C*	1.04
	2	20637563	rs81355894	20,417,294 – 21,634,205	*LRRC4C*	1.04
	2	20665892	rs81355903	20,417,294 – 21,634,205	*LRRC4C*	1.04
	2	20717076	rs81355915	20,417,294 – 21,634,205	*LRRC4C*	1.04
	6	70313133	rs81320475	70,313,309–70,326,455	*RBP7*	3.36
	6	70323076	rs81285644	70,313,309–70,326,455	*RBP7*	3.36
	6	70408106	rs81275494	70,345,801–70,462,430	*UBE4B*	3.36
	6	70418172	rs81279050	70,345,801–70,462,430	*UBE4B*	3.36
	6	70428427	rs81270030	70,345,801–70,462,430	*UBE4B*	3.36
	14	8994023	rs80863686	8,990,750–8,997,300	*NEFL*	1.04
	14	9071879	rs80807276	74.57 kb upstream gene	*NEFL*	1.04
BAL	2	20563683	rs81291755	20,417,294 – 21,634,205	*LRRC4C*	1.56
	2	20637563	rs81355894	20,417,294 – 21,634,205	*LRRC4C*	1.56
	2	20665892	rs81355903	20,417,294 – 21,634,205	*LRRC4C*	1.56
	2	20717076	rs81355915	20,417,294 – 21,634,205	*LRRC4C*	1.56
	2	125660328	rs81265647	3.09 kb upstream gene	*ZNF474*	1.06
	6	70313133	rs81320475	70,313,309–70,326,455	*RBP7*	3.06
	6	70323076	rs81285644	70,313,309–70,326,455	*RBP7*	3.06
	6	70408106	rs81275494	70,345,801–70,462,430	*UBE4B*	3.06
	6	70418172	rs81279050	70,345,801–70,462,430	*UBE4B*	3.06
	6	70428427	rs81270030	70,345,801–70,462,430	*UBE4B*	3.06
	6	168897980	rs81345088	168,876,871–168,902,812	*ZMYND12*	1.08
	6	168899114	rs81259198	168,876,871–168,902,812	*ZMYND12*	1.08
	6	168916590	rs81245903	3.17 kb downstream gene	*RIMKLA*	1.08
	6	168931165	rs81273774	168,919,762–168,950,094	*RIMKLA*	1.08
W2CL	2	107889586	rs81362373	2.18 kb upstream gene	*SLCO4C1*	1.11
	2	107984868	rs81362382	107,922,002–108,020,311	*SLCO6A1*	1.09
	2	108025814	rs81362385	5.50 kb upstream gene	*SLCO6A1*	1.09
	2	108070963	rs81245337	50.65 kb upstream gene	*SLCO6A1*	1.23
	6	70313133	rs81320475	70,313,309–70,326,455	*RBP7*	5.69
	6	70323076	rs81285644	70,313,309–70,326,455	*RBP7*	5.69
	6	70408106	rs81275494	70,345,801–70,462,430	*UBE4B*	5.69
	6	70418172	rs81279050	70,345,801–70,462,430	*UBE4B*	5.69
	6	70428427	rs81270030	70,345,801–70,462,430	*UBE4B*	5.69
	X	7098283	rs80897515	80.54 kb upstream gene	*CLCN4*	1.57
	X	7106161	rs324666200	88.42 kb upstream gene	*CLCN4*	1.42
	X	7116982	rs80818513	99.24 kb upstream gene	*CLCN4*	1.42
W2CL	X	7213512	rs80991855	24.42 downstream gene	*MID1*	1.42
LSY	2	107889586	rs81362373	2.18 kb upstream gene	*SLCO4C1*	1.30
	2	107984868	rs81362382	107,922,002–108,020,311	*SLCO6A1*	1.31
	2	108025814	rs81362385	5.50 kb upstream gene	*SLCO6A1*	1.32
	2	108055321	rs81267184	35.01 kb upstream gene	*SLCO6A1*	1.34
	2	108070963	rs81245337	50.65 kb upstream gene	*SLCO6A1*	2.05
	X	7106161	rs324666200	88.42 kb upstream gene	*CLCN4*	1.38
	X	7116982	rs80818513	99.24 kb upstream gene	*CLCN4*	1.38
	X	7213512	rs80991855	24.42 downstream gene	*MID1*	1.38
PWSY	2	108070963	rs81245337	50.65 kb upstream gene	*SLCO6A1*	1.26
	6	70313133	rs81320475	70,313,309–70,326,455	*RBP7*	1.67
	6	70323076	rs81285644	70,313,309–70,326,455	*RBP7*	1.67
	6	70408106	rs81275494	70,345,801–70,462,430	*UBE4B*	1.67
	6	70418172	rs81279050	70,345,801–70,462,430	*UBE4B*	1.67
	6	70428427	rs81270030	70,345,801–70,462,430	*UBE4B*	1.67
NPD	2	107889586	rs81362373	2.18 kb upstream gene	*SLCO4C1*	1.39
	2	107984868	rs81362382	107,922,002–108,020,311	*SLCO6A1*	1.41
	2	108025814	rs81362385	5.50 kb upstream gene	*SLCO6A1*	1.42
	2	108055321	rs81267184	35.01 kb upstream gene	*SLCO6A1*	1.44
	2	108070963	rs81245337	50.65 kb upstream gene	*SLCO6A1*	2.20
	X	7213512	rs80991855	24.42 downstream gene	*MID1*	1.37
	X	7116982	rs80818513	99.24 kb upstream gene	*CLCN4*	1.37
	X	7106161	rs324666200	88.42 kb upstream gene	*CLCN4*	1.37

SNP, single nucleotide polymorphism; SSC, the position of SNP on *Sus scrofa* chromosome; PWL, pigs weaned per litters; LRRC4C, leucine rich repeat containing 4C; *RBP7*, retinol binding protein 7; *UBE4B*, ubiquitination factor E4B; *NEFL*, neurofilament light; BAL, born alive per litters; *ZNF474*, zinc finger protein 474; *ZMYND12*, zinc finger MYND-type containing 12; *RIMKLA*, ribosomal modification protein rimK like family member A; W2CL, wean to conception interval per litters; *MID1*, midline-1; LSY, litter per sow per year; *SLCO4C1*, solute carrier organic anion transporter family member 4C1; *CLCN4*, chloride voltage-gated channel 4; PWSY, pig weaned per sow per year; NPD, non-productive day.

1) Gene locations on the *Sus scrofa* Build 11.1 assembly and the upstream and downstream of regions that possibly associated with each reproduction traits. Gene names represent on Ensembl (http://asia.ensembl.org/Sus_scrofa/Info/Index).

2) Percentage of genetic variance explained by windows of 5 adjacent SNPs.

**Table 4:** The regions of 5 SNP windows which explained >1% of genetic variance for six reproduction traits in Large White, with a list of annotated genes

Traits	SSC	SNP position	Reference SNP ID number	Gene location1) (bp)	Candidate gene1)	%(var)2)
PWL	1	139481542	rs80830052	139,450,945–139,492,015	*ALDH1A3*	4.27
	1	139579572	rs80930659	139,494,121–139,624,607	*LRRK1*	4.24
	1	139608452	rs80804265	139,494,121–139,624,607	*LRRK1*	4.24
	1	139636710	rs80862569	12.10 kb upstream gene	*LRRK1*	4.24
	1	139655026	rs80846651	30.41 kb upstream gene	*LRRK1*	4.24
	X	91880535	rs81473442	91,724,620–91,880,064	*TRPC5*	1.24
	X	92070342	rs81323503	91,920,451–92,333,326	*RTL4*	1.65
	X	92244402	rs81283192	91,920,451–92,333,326	*RTL4*	1.64
	X	92330719	rs337547716	91,920,451–92,333,326	*RTL4*	1.61
	X	92447181	rs80834138	92,418,941–92,486,688	*LHFPL1*	1.47
BAL	1	139481542	rs80830052	139,450,945–139,492,015	*ALDH1A3*	4.75
	1	139579572	rs80930659	139,494,121–139,624,607	*LRRK1*	4.79
	1	139608452	rs80804265	139,494,121–139,624,607	*LRRK1*	4.80
	1	139636710	rs80862569	12.10 kb upstream gene	*LRRK1*	4.83
	1	139655026	rs80846651	30.41 kb upstream gene	*LRRK1*	4.86
	X	91880535	rs81473442	91,724,620–91,880,064	*TRPC5*	1.32
	X	92070342	rs81323503	91,920,451–92,333,326	*RTL4*	1.76
	X	92244402	rs81283192	91,920,451–92,333,326	*RTL4*	1.76
	X	92330719	rs337547716	91,920,451–92,333,326	*RTL4*	1.76
	X	92447181	rs80834138	92,418,941–92,486,688	*LHFPL1*	1.62
	X	118854990	rs81339510	20.04 upstream gene	*SLITRK2*	1.01
W2CL	1	139481542	rs80830052	139,450,945–139,492,015	*ALDH1A3*	3.52
	1	139579572	rs80930659	139,494,121–139,624,607	*LRRK1*	3.65
	1	139608452	rs80804265	139,494,121–139,624,607	*LRRK1*	3.65
	1	139636710	rs80862569	12.10 kb upstream gene	*LRRK1*	3.66
	1	139655026	rs80846651	30.41 kb upstream gene	*LRRK1*	3.66
	X	113854897	rs80912014	113,460,191–113,955,691	*FGF13*	0.99
	X	113870858	rs80968752	113,460,191–113,955,691	*FGF13*	0.99
	X	113889934	rs80865791	113,460,191–113,955,691	*FGF13*	0.99
	X	113913461	rs80827323	113,460,191–113,955,691	*FGF13*	0.99
	X	118839264	rs328629988	4.31 upstream gene	*SLITRK2*	2.72
	X	118854990	rs81339510	20.04 upstream gene	*SLITRK2*	2.99
LSY	1	139481542	rs80830052	139,450,945–139,492,015	*ALDH1A3*	1.32
	1	139579572	rs80930659	139,494,121–139,624,607	*LRRK1*	1.37
	1	139608452	rs80804265	139,494,121–139,624,607	*LRRK1*	1.39
	1	139655026	rs80846651	30.41 kb upstream gene	*LRRK1*	1.45
	1	139636710	rs80862569	12.10 kb upstream gene	*LRRK1*	1.43
LSY	18	7404576	rs81467652	7,346,793–7,419,859	*EPHB6*	1.00
	X	91880535	rs81473442	91,724,620–91,880,064	*TRPC5*	1.27
	X	92070342	rs81323503	91,920,451–92,333,326	*RTL4*	1.64
	X	92244402	rs81283192	91,920,451–92,333,326	*RTL4*	1.62
	X	92330719	rs337547716	91,920,451–92,333,326	*RTL4*	1.59
	X	92447181	rs80834138	92,418,941–92,486,688	*LHFPL1*	1.45
PWSY	1	139481542	rs80830052	139,450,945–139,492,015	*ALDH1A3*	1.30
	1	139579572	rs80930659	139,494,121–139,624,607	*LRRK1*	1.27
	1	139608452	rs80804265	139,494,121–139,624,607	*LRRK1*	1.26
	1	139636710	rs80862569	12.10 kb upstream gene	*LRRK1*	1.25
	1	139655026	rs80846651	30.41 kb upstream gene	*LRRK1*	1.24
	X	91880535	rs81473442	91,724,620–91,880,064	*TRPC5*	1.43
	X	92070342	rs81323503	91,920,451–92,333,326	*RTL4*	1.86
	X	92244402	rs81283192	91,920,451–92,333,326	*RTL4*	1.85
	X	92330719	rs337547716	91,920,451–92,333,326	*RTL4*	1.82
	X	92447181	rs80834138	92,418,941–92,486,688	*LHFPL1*	1.67
NPD	1	139481542	rs80830052	139,450,945–139,492,015	*ALDH1A3*	1.24
	1	139579572	rs80930659	139,494,121–139,624,607	*LRRK1*	1.29
	1	139608452	rs80804265	139,494,121–139,624,607	*LRRK1*	1.30
	1	139655026	rs80846651	30.41 kb upstream gene	*LRRK1*	1.36
	1	139636710	rs80862569	12.10 kb upstream gene	*LRRK1*	1.34
	18	7404576	rs81467652	7,346,793–7,419,859	*EPHB6*	1.10
	X	7310670	rs81473166	7,237,938–7,421,430	*MID1*	1.06
	X	7483667	rs80838369	7,235,386–7,906,049	*MID1*	1.04
	X	7576417	rs81473214	7,235,386–7,906,049	*MID1*	1.05
	X	7702300	rs81473219	7,235,386–7,906,049	*MID1*	1.05
	X	91880535	rs81473442	91,724,620–91,880,064	*TRPC5*	1.15
	X	92070342	rs81323503	91,920,451–92,333,326	*RTL4*	1.44
	X	92244402	rs81283192	91,920,451–92,333,326	*RTL4*	1.43
	X	92330719	rs337547716	91,920,451–92,333,326	*RTL4*	1.40
	X	92447181	rs80834138	92,418,941–92,486,688	*LHFPL1*	1.30
	X	118275204	rs81473813	173.66 kb downstream	*Uncharacterized*	1.57

SNP, single nucleotide polymorphism; SSC, the position of SNP on *Sus scrofa* chromosome; PWL, pigs weaned per litters; *ALDH1A3*, aldehyde dehydrogenase 1 family member A3; *LRRK1*, leucine rich repeat kinase 1; *TRPC5*, transient receptor potential cation channel subfamily C member 5; *RTL4*, retrotransposon Gag like 4; *LHFPL1*, LHFPL tetraspan subfamily member 1; BAL, born alive per litters; *SLITRK2*, SLIT and NTRK like family member 2; W2CL, wean to conception interval per litters; *FGF13*, fibroblast growth factor 13; LSY, litter per sow per year; *EPHB6*, EPH receptor B6; PWSY, pig weaned per sow per year; NPD, non-productive day; *MID1*, midline-1.

1) Gene locations on the *Sus scrofa* Build 11.1 assembly and the upstream and downstream of regions that possibly associated with each reproduction traits. Gene names represent on Ensembl (http://asia.ensembl.org/Sus_scrofa/Info/Index).

2) Percentage of genetic variance explained by windows of 5 adjacent SNPs.

**Table 5:** The summary of candidate genes overlap in each reproduction trait

Breeds	SSC	Gene1)	Location (bp)	Traits
LR	2	*LRRC4C*	20,417,294 – 21,634,205	PWL and BAL
	2	*SLCO4C1*	107,817,177–107,887,399	W2C, LSY, and NPD
	2	*SLCO6A1*	107,922,002–108,020,311	W2C, LSY, PWSY, and NPD
	2	*ZNF474*	125,663,425–125,719,312	BAL
	6	*RBP7*	70,313,309–70,326,455	PWL, BAL, W2CL, and PWSY
	6	*UBE4B*	70,345,801–70,462,430	PWL, BAL, W2CL, and PWSY
	6	*ZMYND12*	168,876,871–168,902,812	BAL
	6	*RIMKLA*	168,919,762–168,950,094	BAL
	14	*NEFL*	8,990,750–8,997,300	PWL
	X	*CLCN4*	6,977,164–7,017,734	W2C, LSY, and NPD
	X	*MID1*	7,235,386–7,906,049	W2C, LSY, and NPD
LW	1	*ALDH1A3*	139,450,945–139,492,015	Six traits
	1	*LRRK1*	139,494,121–139,624,607	Six traits
	18	*EPHB6*	7,346,793–7,419,859	LSY and NPD
	X	*MID1*	7,235,386–7,906,049	NPD
	X	*RTL4*	91,920,451–92,333,326	PWL, BAL, LSY, PWSY, and NPD
	X	*TRPC5*	91,724,620–91,880,064	PWL, BAL, LSY, PWSY, and NPD
	X	*LHFPL1*	92,418,941–92,486,688	PWL, BAL, LSY, PWSY, and NPD
	X	*SLITRK2*	118,826,442–118,834,950	BAL and W2CL
	X	*FGF13*	113,460,191–113,955,691	W2CL

SSC, the position of SNP on Sus scrofa chromosome; LR, Landrace; *LRRC4C*, leucine rich repeat containing 4C; PWL, pigs weaned per litters; BAL, born alive per litters; *SLCO4C1*, solute carrier organic anion transporter family member 4C1; W2CL, wean to conception interval per litters; LSY, litter per sow per year; PWSY, pig weaned per sow per year; NPD, non-productive day; *ZNF474*, zinc finger protein 474; *RBP7*, retinol binding protein 7; *UBE4B*, ubiquitination factor E4B; *ZMYND12*, zinc finger MYND-type containing 12; *RIMKLA*, ribosomal modification protein rimK like family member A; *NEFL*, neurofilament light; *CLCN4*, chloride voltage-gated channel 4; *MID1*, midline-1; LW, Large White; *ALDH1A3*, aldehyde dehydrogenase 1 family member A3; *LRRK1*, leucine rich repeat kinase 1; *EPHB6*, EPH receptor B6; *RTL4*, retrotransposon Gag like 4; *TRPC5*, transient receptor potential cation channel subfamily C member 5; *LHFPL1*, LHFPL tetraspan subfamily member 1; *SLITRK2*, SLIT and NTRK like family member 2; *FGF13*, fibroblast growth factor 13.

1) Gene locations on the *Sus scrofa* Build 11.1 assembly. Gene names represent on Ensembl (http://asia.ensembl.org/Sus_scrofa/Info/Index).
